# Closed-Loop Medication Management with an Electronic Health Record System in U.S. and Finnish Hospitals

**DOI:** 10.3390/ijerph20176680

**Published:** 2023-08-30

**Authors:** Susan B. Shermock, Kenneth M. Shermock, Lotta L. Schepel

**Affiliations:** 1 Howard County Medical Center, The Johns Hopkins Health System, Department of Pharmacy Services, 5755 Cedar Lane, Columbia, MD 21044, USA; sshermock@jhmi.edu; 2 Center for Medication Quality and Outcomes, The Johns Hopkins Health System, 600 North Wolfe Street Carnegie 180, Baltimore, MD 21287, USA; ken@jhmi.edu; 3 Division of Pharmacology and Pharmacotherapy, Faculty of Pharmacy, University of Helsinki, 00029 Helsinki, Finland; 4 Quality and Patient Safety Unit and HUS Pharmacy, HUS Joint Resources, Helsinki University Hospital and University of Helsinki, 00029 Helsinki, Finland

**Keywords:** medication safety, medication error, closed-loop, electronic medication management system, electronic health record, computerized provider order entry, clinical decision support system, barcode assisted medication administration, automated dispensing cabinet, EPIC

## Abstract

Many medication errors in the hospital setting are due to manual, error-prone processes in the medication management system. Closed-loop Electronic Medication Management Systems (EMMSs) use technology to prevent medication errors by replacing manual steps with automated, electronic ones. As Finnish Helsinki University Hospital (HUS) establishes its first closed-loop EMMS with the new Epic-based Electronic Health Record system (APOTTI), it is helpful to consider the history of a more mature system: that of the United States. The U.S. approach evolved over time under unique policy, economic, and legal circumstances. Closed-loop EMMSs have arrived in many U.S. hospital locations, with myriad market-by-market manifestations typical of the U.S. healthcare system. This review describes and compares U.S. and Finnish hospitals’ EMMS approaches and their impact on medication workflows and safety. Specifically, commonalities and nuanced differences in closed-loop EMMSs are explored from the perspectives of the care/nursing unit and hospital pharmacy operations perspectives. As the technologies are now fully implemented and destined for evolution in both countries, perhaps closed-loop EMMSs can be a topic of continued collaboration between the two countries. This review can also be used for benchmarking in other countries developing closed-loop EMMSs.

## 1. Introduction

A medication error (ME) is a preventable event that may cause or lead to inappropriate medication use or patient harm [1]. MEs are the leading preventable factor jeopardizing patient safety and, globally, the annual cost associated with MEs has been estimated at USD 42 billion [2]. MEs occur when weak medication management systems and human factors such as fatigue and staff shortages affect healthcare delivery [2]. The processes of medication prescribing, administering, and monitoring, and transitions of care have been identified as the most error-prone activities within the medication use system [2,3,4].

**Figure 1:**
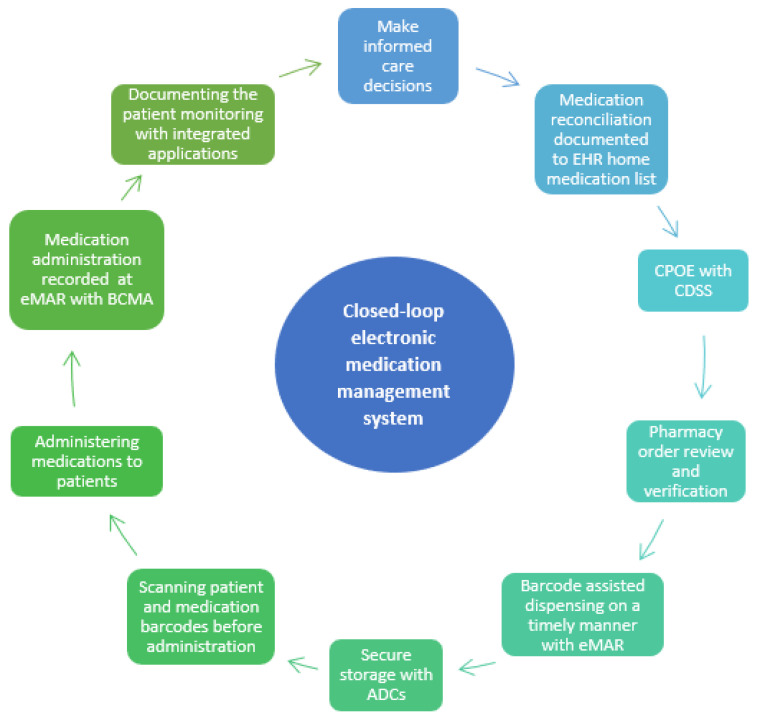
Closed-loop electronic medication management system on nursing/care units (EMMS, adapted from [11]. CPOE = Computerized Physician Order Entry; CDSS = Clinical Decision Support System; eMAR = Electronic Administration Record; ACD = Automated Dispensing Cabinet. BCMA = Barcoded Medication Administration.

Closed-loop Electronic Medication Management Systems (EMMSs) are seen as potential technological solutions to prevent MEs [5,6,7,8]. Electronic medication management refers to a closed-loop system that encompasses prescribing, pharmacy verification, smart infusion pumps, automated dispensing cabinets, barcoded medication administration (BCMA), and anything that has electronic or digital medicine datasets or encompasses medication management processes (Figure 1) [5,9].

The aim of using a closed-loop technology approach is to decrease the manual, error-prone human labor in the medication management process (e.g., verbal, handwritten orders, or manual double checks). Electronic health record (EHR) systems should enable this technology to achieve a closed-loop medication management process with EMMSs [10]. However, new technology can introduce new challenges and processes that need to be managed [10]. Furthermore, new technology is usually expensive and its value and effects on patient safety, quality, and resource management should be carefully considered.

Hospitals in the United States (U.S.) have been leaders in implementing EMMSs as incremental adoption has occurred over nearly 50 years [12]. Incentives for integrating EMMSs in hospitals come from government regulations, litigation avoidance, and ratings by independent organizations. EMMS technology is desired to improve the efficiency, safety, and quality of healthcare [13,14,15,16,17,18]. Conversely, Helsinki University Hospital (HUS) implemented the Epic-based APOTTI EHR system, which enables the implementation of a closed-loop EMMS, as recently as 2018–2021 [10]. HUS is the first Finnish hospital to introduce a closed-loop EMMS [10]. The aim of this review is to describe and compare U.S. and Finnish hospitals’ EMMS approaches and their impact on medication safety and workflows. This review can be used for benchmarking in other countries developing closed-loop EMMSs.

## 2. Closed-Loop EMMSs in U.S. and Finnish Hospitals

### 2.1. Development of Closed-Loop EMMSs in U.S. Hospitals

In the U.S., hospital care is provided by a mix of federal, state, private, and independent entities [19]. U.S. hospitals need “deemed” status based on individual hospital surveys of quality care to meet ongoing federal requirements for reimbursement for care of citizens who qualify for U.S. federal health insurance (the poor, disabled, or elderly) [20]. In the 1970s, the American Society of Health System Pharmacists (ASHP) supported the use of unit-dose medications [12]. Automated dispensing cabinets first became available in the 1980s [21]. Barcode Medication Administration and Physician Order Entry were introduced in the 1990s [22,23]. Interest in improving medication safety using these tools increased with the Institute of Medicine report “To Err is Human” [24]. Adoption of EMMSs significantly increased with the introduction of the U.S. federal law, the Health Information Technology for Economic and Clinical Health Act (HITECH), included in the 2009 stimulus package. This law incentivized the adoption of an EHR by hospitals to meet ‘meaningful use’ standards, which uses financial rewards and penalties of government reimbursement for the care of citizens who qualify for U.S. federal health insurance [13,14]. ‘Meaningful use’ required the development of a health information exchange to facilitate communication information across technologies and healthcare providers to make care safer and more efficient [15].

The key elements in the U.S. system for care/nursing units and hospital pharmacies are presented in Table 1 and Table 2. The implementation and ongoing utilization of EMMSs is multidisciplinary. While many clinical services interact with and may suggest improvements to a hospital’s EMMS (e.g., medical staff, nursing, pharmacy, respiratory therapy, laboratory/phlebotomy, diagnostic imaging), EMMSs require the involvement of facilities, informatics, clinical engineering, and human factors engineers to be successful [25]. Special attention must be paid to areas of high-risk care or the use of high-alert medications in areas such as oncology, pediatrics/neonates, or operative areas [26,27,28,29].

### 2.2. Helsinki University Hospital Introduced Closed-Loop EMMSs to Finland

The Finnish healthcare system is a public system funded by the government and operated by regional well-being services counties. The private sector complements public care. Helsinki University Hospital (HUS) provides public secondary and tertiary care via 23 hospitals with approximately 3000 beds for a population of 1.6 million in the capital area of Finland. HUS implemented an Epic-based EHR system (APOTTI) in four phases during 2018–2021 [10] alongside several counties providing primary and social care in the capital area of Finland. The APOTTI supports, for the first time in Finland, a closed-loop EMMS in the hospital setting [10]. The Ministry of Social Affairs and Health encourages Finnish hospitals to implement EMMSs, but it is not mandatory [30].

In addition to several municipalities providing primary and social care in the capital area of Finland, all 23 HUS tertiary care hospitals and clinical areas are using APOTTI [10] so there is only one EHR and ordering system at HUS. Prior to the introduction of APOTTI, there were multiple systems in use between the hospitals and even in different care units within one hospital (e.g., different systems in the emergency department, operating theaters, and nursing/care units) that were not integrated. The key elements of HUS’s current system are presented in Table 1 and Table 2. However, the HUS hospital pharmacy has its own system for purchasing, storing, inventory, dispensing, and medication preparation (Table 2), which is integrated with EHR (HUS enterprise resource planning (ERP)). The previous HUS EHR system did not include crucial elements of an EMMS (Figure 1). Hence, implementing APOTTI required major process changes [10]. For example, medication reconciliation did not have a technical workflow with Kanta integration (which holds electronic prescriptions) in the earlier EHR system, home medications were documented with free text, limiting integration options, and pharmacists were not widely involved.

**Table 1:** Closed-loop electronic medication management process on nursing/care units in the U.S. and Finland. ACD = Automated Dispensing Cabinet. BCMA = Barcoded Medication Administration, eMAR = Electronic Administration Record; HAM = High-Alert Medication [31].

United States (Hospitals with 200 or More Beds)	Helsinki University Hospital, Finland [10]
**Medication Reconciliation: home medication list obtained using two sources**	
Medication reconciliation and nursing or pharmacy staff obtain the best-possible medication history (prior-to-admission medication lists) and compliance rates are monitored. External medication history information is pulled into the EHR from outside sources such as retail pharmacies. Pharmacists and pharmacy technicians are frequently involved [32,33,34].	Medication reconciliation and nursing or pharmacy staff obtain the best-possible medication history (prior-to-admission medication lists) and compliance rates are monitored. Medication reconciliation and a structured home medication list are mandatory for in-patient medication. The EHR home medication list is integrated into the national Kanta system [35], which holds electronic prescriptions. Pharmacists are involved in many units.
**Ordering/prescribing with computerized physician order entry (CPOE)**	
Provides ordering support, through structured order and prescription forms, for most common doses/frequencies. Order panels and order sets developed for specific diagnoses or situations (e.g., admission) [36].	
**Clinical Decision Support System (CDSS)**	
Sophisticated CDSS, e.g., with dose warnings (including dosing with older patients and renal impairment), duplicate medications, and electronic best practice advice (BPA) [37,38].	
**Dispensing and automated dispensing cabinets (ADCs)**	
ADCs are widely used, integrated with EHR, and enable the dispensing of medicines according to verified electronic orders on many units. Medication removal by override is limited to urgently needed medications (e.g., antidotes, medications for intubation) [39]. ADC overrides display in EMR to be reconciled with prescriber order and allow BCMA. While some barcodes include lot number and expiration date, scanning technology in use is reading a medication’s National Drug Code (NDC) number. Starting in November 2023, barcodes must include lot numbers and expiration dates [40].	ADCs are in use in many units and integrated with EHR, which enables the dispensing of medicines according to electronic orders. Medication removal by override is not yet limited. Nurses do the dispensing in a timely manner (max. 2 h before administration) by using the eMAR and scanning the barcodes of the medicine secondary packages (unit doses are not available yet). Barcodes include a lot number and expiration date [41]. A manual double-check is used when the barcode is not available and for HAMs.
**Preparation outside of the pharmacy**	
On units, intravenous preparation is limited to emergencies, drawing medications into syringes for IV Push or IM administration, or the use of vial and bag adaptor technology [42]. Efforts are made to dispense most medications as ready-to-use and unit-dosed by the hospital pharmacy	Ready-to-use medications are not widely available and preparing is commonly done by nurses or pharmacists. EHR provides the documentation with barcodes and instructions for preparation. The manual double-check is used when the barcode is not available and for HAMs.
**Administration**	
Medication administration is recorded promptly at the bedside using BCMA confirming the right patient, medication, dose, time, and route.	
Most hospitals use smart pumps, some hospitals utilize IV pump interoperability with EHR [39]. High-alert titrated infusion medications may include a MAR calculator to assist with titrations (e.g., heparin or insulin). Some HAMs may require a manual independent double-check process documented in EHR.	IV pump interoperability with EHR is not yet in use.
**Patient Monitoring**	
Interfaced when technology allows. Monitoring data included in dashboards; patient scoring tools, or machine learning used for early identification of diseases such as sepsis and acute kidney injury [43,44,45,46].	Interfaced when technology allows. Monitoring data included in dashboards; patient scoring tools used for early identification of diseases such as sepsis.
**Communication**	
EMR allows for secure electronic instant communication between members of the healthcare team using secure instant messaging. Order communication between pharmacy and nurse. Follow-up communication between shifts. Epic users have the MyChart phone App for patients to read their charts and laboratory results, communicate with healthcare professionals, and report their home medications and allergies, for example.	

**Table 2:** Closed-loop electronic medication management at hospital pharmacies in the U.S. and Finland. Finnish HUS Pharmacy uses its own enterprise resource planning system (ERP) for storing, inventory, and preparation, but it is integrated with the electronic health record system (EHR). ADC = Automated Dispensing Cabinet.

United States	Helsinki University Hospital, Finland [10]
**Pharmacist Medication Order Verification**	
Prospective pharmacist verification for all orders. Exceptions are emergent/urgent medication needs or medication in the presence of a physician [47].	Retrospective pharmacist verification of specific orders (e.g., high-alert medications) in some units during weekdays and after the fact for weekends.
**Purchasing, storing, and inventory**	
Continuous inventory allows for as-needed purchasing and enhanced management of medication shortages facilitated by integration of her, ADCs, and in some hospitals, automated drug storage such as carousels or robots [39].	There is integration between EHR and hospital pharmacy’s ERP regarding ADCs. Information on orders and patients comes from EHR to ERP and doses taken from ADC go to EHR. Storage automation and barcode scanning are in use with the hospital pharmacy’s ERP, which is in use for purchasing, storage, and inventory.
**Dispensing**	
Automated drug storage and retrieval (e.g., carousels or robots) may be used that coordinate patient orders with medication dispensing through an interface [39]. Dispensing and stocking are verified with barcode scanning. Unit-dose dispensing prioritized for medications (exceptions: bulk medications such as creams, ointments, ophthalmic/otic drops, and insulin pens). If not stocked in ADC, first doses are prioritized and sent to units regularly from the main pharmacy. Ongoing scheduled medications are dispensed to units at specified times during the day based on upcoming administration times.	Dispensing is integrated with EHR only regarding ADCs and multidose dispensing, which is in use in primary and social care, where the HUS Pharmacy also dispenses medications. Information on orders and patients comes from EHR to ERP and information on prepared doses (including lot numbers and expiration dates) goes back to EHR. Unit-dose dispensing is not yet in use, but HUS is planning and preparing it for its next new hospital.
**Sterile Medication Preparation**	
Use of barcode scanning of medication and diluent during preparation in sterile preparation facilities connected to order in EHR. Photo documentation and gravimetric confirmation are possible at many hospitals [39]. The final product is provided with a scannable barcode for BCMA.	Integrated into EHR system. Hospital pharmacy prepares patient-specific ready-to-use cytotoxic and biological medications, botulin toxin solutions, and total parenteral nutrition. Information on orders and patients comes from the EHR to the hospital pharmacy’s ERP and information on prepared doses (including lot numbers and expiration dates) goes back to the EHR. Preparation robots including gravimetric confirmation and barcode scanning are in use for cytotoxic medications.
**Communication**	
EHR allows for secure electronic instant communication between members of the healthcare team using secure instant messaging. Order communication between pharmacy and nurse. Follow-up communication between shifts.	

The ordering process was not as structured, CDSS included only allergy and interaction warnings, BCMA was not available, and ADCs were not integrated with the EHR system [10]. Medicines were dispensed in the care/nursing units to cover the next 24 h, instead of just prior to the ordered administration time.

### 2.3. Comparing Closed-Loop EMMSs in U.S. and Finnish Hospitals

Table 1 presents similarities and differences between U.S. and Finnish EMMS activities on nursing/care units. The U.S. and Finland use similar processes for ordering/prescribing with computerized CPOE and provide clinical decision support (CDSS) for the healthcare team. In both countries, medication administration is completed with barcode medication administration (BCMA) and integrated patient monitoring technology, with the EMMS providing interprofessional communication tools. The U.S. is ahead of Finland in the use of smart pumps integrated with EHR (Table 1). Furthermore, hospitals in the U.S. use more unit-dose and ready-to-use medications; hence, BCMA is dose-specific. In Finland, nurses dispense medications to create patient-specific doses and prepare medications on the nursing/care units. Finnish nurses scan barcodes on secondary (bulk) packages instead of primary packages or unit doses. At the hospital pharmacy level, there are more differences (Table 2). In the U.S., the pharmacist medication order verification is done prospectively before medication dispensing and administration. Nurses cannot pull routine medications out from ADCs before orders are verified. In Finland, clinical pharmacy resources are not as widespread, and at this point, in most of Finland, order verification is done retrospectively for limited medications (e.g., high-alert medications) and only on weekdays (Monday to Friday) [10,48]. Nurses can administer medications before verification. However, with the new EHR system (Epic-based APOTTI), HUS is the first hospital in Finland that has been able to start prospective order verification for pharmacists [10]. In EMMSs, order verification is a crucial defensive step because it proceeds administration with BCMA, which relies on correct orders. Another difference is that the Finnish HUS Pharmacy did not implement the Epic system for storage, inventory, and preparation purposes (Table 2). This approach was chosen because the Epic-based EHR system did not fulfill the requirements of the Finnish Act on Stockpiling of Medicine (19 December2008/979) and standards on good manufacturing practices related to medicine preparation regulated by the Finnish Medicines Agency [49,50].

## 3. Functionality of Closed-Loop EMMSs across the Medication Use System

A closed-loop EMMS decreases or eliminates many historic medication management problems associated with human error as it incorporates forcing functions, barriers, fail safes, and automation into many steps in the medication-use process (i.e., medication reconciliation, ordering/prescribing, transcription, purchasing/storage, compounding/ preparation, dispensing, administration, and patient monitoring) [51]. Ideally, an EMMS provides interconnectivity or interfaces between all steps in the medication-use process, allowing for clinical input to be carried out without the need for transcription. An EMMS improves documentation and reporting processes by providing a centralized electronic platform where medication-related information such as administration records, adverse events, and clinical outcomes can be recorded, accessed, and analyzed, which enables knowledge-based management and data-based value creation. Furthermore, as an institution matures in its use of an EHR as a critical component of an EMMS, patient mortality has been shown to decrease [52].

### 3.1. Medication Reconciliation

The medication reconciliation process is streamlined and properly documented with the use of an EMMS. Obtaining accurate medication lists is facilitated by the provision of outpatient prescription information, which can be used with other sources to develop a comprehensive prior-to-admission/home medication list. The use of an EMMS facilitates medication reconciliation during transitions of care, such as hospital admissions, transfers, and discharges, which are high-risk situations from a medication safety perspective [2]. By providing a comprehensive electronic record of a patient’s medication history, the EMMS improves medication accuracy and reduces the risk of medication discrepancies.

In the U.S. and Finland, medication reconciliation requires manually reviewing and obtaining a best-possible prior-to-admission/home medication list using two sources, which is time-consuming (Table 1). Once this process is complete, the EMMS facilitates medication reconciliation during transitions of care [32,33,34]. In Helsinki University Hospital (HUS), the implementation of APOTTI has forced healthcare professionals to conduct medication reconciliation as it should be done because a structured home medication list is used as the basis for an inpatient medication list [10]. In HUS’ earlier EHR system, it was easier to skip this step, which led to major discrepancies in the prior-to-admission/home medication list [53]. With the new EHR system, these errors are no longer hidden, and clinical pharmacists are a widely involved and appreciated resource for conducting medication reconciliations [10,54]. However, this more structured process is laborious, and the nationwide development of the Kanta system holding updated structured home medication lists in addition to electronic outpatient prescriptions [35] should be done urgently to save time and resources without risking medication safety [10].

### 3.2. Ordering/Prescribing with Computerized Physician Order Entry (CPOE) with Clinical Decision Support System (CDSS)

Electronic prescribing reduces the chances of misinterpretation by other healthcare providers, leading to more accurate orders. Risks caused by transcription and illegible handwriting are eliminated [36]. Clinical Decision Support (CDSS) in an EMMS performs real-time checks or provides just-in-time information for potential drug allergies and interactions, renal function information to guide dosing, alerts generated through machine learning, or scoring systems alerting and guiding healthcare professionals to provide optimal care and preventing medication-related complications [37,38,43,44,45].

In HUS as well as in other Nordic countries implementing Epic-based EHR systems, the implementation of a new, structured CPOE has been the “Achilles’ heel” from the EMMS perspective: physicians find Epic more laborious and are not satisfied with its usability [10,55,56,57]. HUS was not sufficiently prepared for this change and physician training for CPOE regarding medication ordering and prescribing was not mandatory, which was clearly a major mistake [10] and has been corrected by defining mandatory training for physicians. After implementing the Epic-based EHR system (APOTTI), ordering and prescribing errors in HUS have increased and become more visible, and were under-reported earlier [10]. In particular, linking orders (different doses for morning and evening or different weekdays) has been very challenging in HUS [10], but Epic’s next Hyperdrive update (coming to HUS in November 2023) is claimed to solve this specific usability difficulty. Furthermore, there is alert fatigue related to new CDSS in HUS, so these alerts need to be optimized [10].

In the U.S., many hospitals have had CPOE with CDSS for several years (Table 1). EHR systems have evolved and matured: they have had many years to refine ordering and alerts in EHR to decrease alert fatigue [58,59] and onboarding of new staff includes training in the use of the EHR system. Currently, there is more concern and preparation for EHR downtimes, when hospitals are forced to return to using paper orders. Scheduled downtimes occur in the middle of the night to limit disruptions in care.

### 3.3. Order Verification

Order verification, a crucial step in an EMMS, is a multistep process during which pharmacists evaluate medication orders for safety and efficacy. Before a medication is dispensed and administered to the patient, including dispensing from an ADC, the pharmacist prospectively reviews and evaluates medication orders for appropriateness. This order verification includes evaluating each order based on patient-dependent factors such as renal function, age, sex, weight, concomitant medications, and allergies; and medication-related factors such as dose, route, frequency, and duration [60,61]. As mentioned previously, U.S. hospitals need “deemed” status based on individual hospital surveys of quality care to meet ongoing federal requirements for reimbursement of care of citizens who qualify for U.S. federal health insurance [20]. Surveyors inspecting hospital care expect prospective pharmacy review of all medication orders with few exceptions. These exceptions are for instances when prospective pharmacy review may not be practical or required. For example, when a patient’s health status is acutely critical and care is required, waiting for pharmacy review could create delays that may result in patient harm [62].

Before implementing the Epic-based EHR (APOTTI) in HUS, there were high hopes for a more advanced clinical decision support system (CDSS) for managing and preventing prescribing errors [10]. There were even discussions that CDSS would replace medication reviews conducted by clinical pharmacists. Pharmacists’ order verification was not in use in Finland earlier, but HUS Pharmacy still wanted to pilot it for high-risk orders (Table 2) and pilot results were promising [48]. After implementing APOTTI, ordering/prescribing errors increased and at the same time, the COVID pandemic accelerated the shortage of nurses. Nowadays, when there are fewer experienced nurses skilled enough to detect prescribing errors before administration, this service is highly appreciated and demanded. Physicians and nurses want order verification to be expanded from weekdays to weekends based on root-cause analysis of severe ordering errors.

### 3.4. Dispensing and Preparing of Medicines

In the U.S., unit-dose medication dispensing is a standard and a cornerstone of the hospital medication distribution system (Table 1). Unit-dose medication packaging has been used in the U.S. to improve patient safety for nearly 50 years [12]. Unit-dose dispensing ensures medications are dispensed and administered from a single unit or unit-dose package as distributed by the pharmacy. The only exceptions are bulk products that are difficult to unit dose (e.g., creams/ointments, eye drops, and insulin pens). Medications are dispensed in as close to a ready-to-administer form as possible. In the case of hospitals with more than 200 beds, many hospitals use automated drug storage and retrieval systems (e.g., carousels or robots) that use barcode scanning and will coordinate both patient orders with medication dispensing and ADC refills through interfaces [39]. Most hospitals with over 200 beds prioritize the use of ADCs on medication units so that nurses can obtain needed doses at the time of administration [39]. These ADCs are often also interfaced with the EHR, allowing the removal of ordered and verified medications only. Medications that may be urgently needed, such as antidotes or medications needed for an acute patient intubation, are available through override from an ADC. For medications not stocked in ADCs, pharmacies dispense no more than a 24 h supply of patient-specific doses to be stored securely and in a patient-specific manner on the units.

When commercially available, sterile medications (e.g., infusions) are purchased ready-to-use as single doses or standard concentrations. Vial-to-bag technology may also be used when appropriate [42]. Most other intravenous preparation is completed in the sterile clean room suite. Barcode scanning against the EHR order of both medication and diluent improves safety. Some hospitals also have technology that provides photo documentation of the compounding process and/or gravimetric confirmation of the final product [39]. The final product is provided with a scannable barcode for BCMA.

In HUS, the dispensing and preparation of medications is still mainly conducted on nursing units (Table 1 and Table 2), because unit doses are not yet available and ready-to-use medicines are limited. Barcode-assisted dispensing and preparing in a timely manner has decreased dispensing errors and replaced the need for time-consuming manual double-checks [10]. However, compliance with using barcodes and following the EHR-guided process is not yet at the optimal level and needs to be monitored and further developed.

### 3.5. Barcoded Medication Administration (BCMA), Electronic Medication Administration Records (eMAR), and Integrated Smart Pumps

The utility of BCMA has been recognized in previous studies [6,7,63]. Electronic medication administration records (eMAR) are clear, standardized, and current compared to paper-based MARs. An EMMS, using BCMA, reduces the chances of incorrect medication administration or missed/duplicate medication administrations, and the need for manual double checks is reduced when BCMAs are used at the primary package level (e.g., unit doses and ready-to-use medications). The use of an EMMS ensures accurate and timely medication administration records. Barcode scanning percentages and compliance levels can be monitored at the hospital, unit, and individual healthcare professional levels, which helps in identifying compliance problems [64]. In HUS, administration errors have noticeably decreased after implementing the Epic-based APOTTI [10].

Some hospitals in the U.S. have developed medication administration record (MAR) calculators to assist with titrations of high-alert medication infusions (e.g., heparin or insulin, Table 1). MAR calculators guide nurses on appropriate infusion dose changes based on monitoring and have improved titration protocol compliance, increased time spent in the therapeutic dose range, and provided increased standardization with less variability compared to non-ERH-integrated protocols [65].

In the U.S., most hospitals use smart pumps [39], which use dose error reduction software (DERS, Table 1). DERS is comprised of a medication library with individualized soft and hard min. and max. doses, rates, durations and/or concentrations for continuous infusions, bolus doses, intermittent infusions, patient-controlled analgesia (PCA), and epidural infusions. The use of DERS guides the safe administration of medications administered. Some hospitals now also utilize IV pump interoperability with their EHR. IV Pump interoperability facilitates the automatic programming of the smart IV pump with the prescriber’s ordered infusion parameters that have been prospectively verified by the pharmacist when the nurse completes BCMA and then scans the pump. This interoperability is bidirectional, so infusion data is automatically documented in the patient’s EHR during administration [66]. HUS is planning to integrate smart pumps into APOTTI and the process of creating a HUS drug library with dose limits has started [67].

### 3.6. Patient Monitoring

Both HUS and the U.S. have monitoring devices interfaced with EHR when technology allows, enabling results to populate appropriate areas of the EHR automatically (Table 1). An example of a point-of-care technology that is integral to EMMS use is glucometers to support diabetes management, which allows real-time clinical decision-making for insulin management [68]. Monitoring data is frequently included in EHR order entry, dashboards, and patient scoring tools to provide just-in-time information and trending or scoring tools to identify patients in need of clinical intervention. Machine learning has also been integrated into EHR for the early identification of diseases such as sepsis and acute kidney injury [43,44,45,46].

### 3.7. Inventory and Stockpiling

Inventory Management utilizing EMMS can enable efficient inventory management by tracking medication usage, lot numbers, expiration dates, and stock levels. This reduces wastage, optimizes supply, assists with drug shortage management (easier to locate medicines on the care/nursing units), and ensures that medications are readily available when and where they are needed. In the U.S., while some barcodes include the lot number and expiration date, the scanning technology currently in use is restricted to reading a medication’s National Drug Code (NDC) number (Table 2). Starting from November 2023, medication barcodes must include lot numbers and expiration dates [40]. In the case of HUS, barcodes include the medication’s lot number and expiration date [41] allowing hospital staff to be alerted when scanning an expired medication (Table 2). HUS has been able to integrate EHR and ADCs but is still using its own ERP for inventory and stockpiling in its pharmacy areas.

### 3.8. Communication with Healthcare Colleagues and Patients

Communication of patient information is simplified, streamlined, and electronically accessible in an EMMS. Healthcare professionals utilizing an EMMS have access to secure messaging tools to communicate and coordinate care across healthcare disciplines and departments. Epic users have the MyChart phone App or web-based application for patients to read their charts and laboratory results, communicate with healthcare professionals, and report their home medications and allergies, for example (Table 1), which has been very popular among HUS patients. The ability to communicate with patients was one of the requirements when HUS decided to adopt a new EHR system.

## 4. Future Directions of EMMSs in the U.S. and Finland

### 4.1. Existing Challenges

Enhancements to EMMSs in the U.S. in the future are most likely to include machine learning and artificial intelligence. Academic hospitals continue to experiment with these technologies, but it has been observed that not all of these innovations are being subjected to clinical trials to demonstrate improvements in patient care [69]. CDSS around medication therapy for older adults, polypharmacy, and opioid safety are areas recently identified where these technologies can have an impact on EMMSs [70]. Introducing a national structured format for patients’ prior-to-admission/home medication lists that could be maintained by outpatient pharmacies, prescribers, and patients would increase the safety and efficiency of our medication reconciliation processes. Lastly, introducing scanning technology that assists with inventory expiration date management could decrease the need for manual checks.

HUS is following U.S. and international developments and is planning to implement unit-dose dispensing and provide more ready-to-use medicines in the future, which should save nursing resources and increase medication safety. Also, interoperability between EHR and infusion pumps should be considered. Furthermore, the CDSS system within the Epic-based Apotti needs optimizing because of existing alert fatigue [10], and a development project related to this has begun. Generally, the medication management parts of Apotti need evolution and maturation from user and usability perspectives, especially related to ordering and prescribing phases [10]. On the national level, the developments of the Kanta system [35] are urgently needed to ease the work related to medication reconciliation (updated structured medication lists integrated with EHRs instead of just holding e-prescriptions). Other hospitals in Finland are following HUS’ steps and are developing and implementing new EHR systems that will enable the integration of EMMS technology, which is already in use in several hospitals without EHR integration.

### 4.2. Opportunities and Application Prospects

Although barcode scanning is beneficial, it is sometimes laborious. Hence, visual scanning [71] instead of barcode scanning is an interesting approach that should be further assessed. Furthermore, artificial intelligence and machine learning are potential technologies to prevent adverse drug events and these have been already applied, e.g., for drug discovery and pharmacovigilance purposes [72,73]. Machine learning is integrated into some hospital EHRs in the U.S. for early identification of diseases such as sepsis and acute kidney injury [43,44,45,46]. Artificial intelligence is being investigated to improve patients’ self-administration of medications [74]. Their potential has also been recognized from medication safety and EMMS perspectives [75] and the first practical tools have been created within CDSS to prevent prescribing errors [76]. As mentioned, U.S. academic hospitals are already experimenting with artificial intelligence, but it has been observed that not all of these innovations are being subjected to clinical trials to demonstrate improvements in patient care [69].

## 5. Conclusions

The use of a closed-loop EMMS decreases or eliminates many historic medication management problems associated with human error as it incorporates forcing functions, barriers, fail safes, and automation into many steps in the medication-use process. Ideally, an EMMS provides interconnectivity or interfaces between all steps in the medication-use process, allowing for clinical input to be done without the need for transcription. However, implementing and using new technology also introduces new errors that need to be managed. Achieving the highest benefits with an EMMS takes time and depends on healthcare professional adoption of the EHR system and the evolution and maturity of EHR systems that comes from the implementation of continuous performance improvement programs after implementation.

## Data Availability

Data are contained within the article.
